# *mcr-1* colistin resistance gene sharing between *Escherichia coli* from cohabiting dogs and humans, Lisbon, Portugal, 2018 to 2020

**DOI:** 10.2807/1560-7917.ES.2022.27.44.2101144

**Published:** 2022-11-03

**Authors:** Juliana Menezes, Joana Moreira da Silva, Sian-Marie Frosini, Anette Loeffler, Scott Weese, Vincent Perreten, Stefan Schwarz, Luís Telo da Gama, Andreia Jesus Amaral, Constança Pomba

**Affiliations:** 1 Centre for Interdisciplinary Research in Animal Health (CIISA), Faculty of Veterinary Medicine, University of Lisbon, Lisbon, Portugal; 2 Associate Laboratory for Animal and Veterinary Sciences (AL4AnimalS), Lisbon, Portugal; 3 Royal Veterinary College, Hertfordshire, United Kingdom; 4 Ontario Veterinary College, Guelph, Ontario, Canada; 5 Institute of Veterinary Bacteriology, Vetsuisse Faculty, University of Bern, Bern, Switzerland; 6 Centre for Infection Medicine, Department of Veterinary Medicine, Institute of Microbiology and Epizootics, Freie Universität Berlin, Berlin, Germany

**Keywords:** Plasmid-mediated colistin resistance, *mcr*, multidrug resistance, *Escherichia coli* ST744, colonisation, companion animals, healthy humans

## Abstract

**Background:**

The emergence of colistin resistance is a One Health antimicrobial resistance challenge worldwide. The close contact between companion animals and humans creates opportunities for transmission and dissemination of colistin-resistant bacteria.

**Aim:**

To detect potential animal reservoirs of colistin-resistant *Escherichia coli* and investigate the possible sharing of these bacteria between dogs, cats and their cohabiting humans in the community in Lisbon, Portugal.

**Methods:**

A prospective longitudinal study was performed from 2018 to 2020. Faecal samples from dogs and cats either healthy or diagnosed with a skin and soft tissue or urinary tract infection, and their cohabiting humans were screened for the presence of colistin-resistant *E*. *coli.* All isolates were tested by broth microdilution against colistin and 12 other antimicrobials. Colistin-resistant isolates were screened for 30 resistance genes, including plasmid-mediated colistin resistance genes (*mcr-1* to *mcr-9*), and typed by multilocus sequence typing. Genetic relatedness between animal and human isolates was analysed by whole genome sequencing.

**Results:**

Colistin-resistant *E. coli* strains harbouring the *mcr-1* gene were recovered from faecal samples of companion animals (8/102; 7.8%) and humans (4/125; 3.2%). No difference between control and infection group was detected. Indistinguishable multidrug-resistant *E*. *coli* ST744 strains harbouring the *mcr-1* gene were found in humans and their dogs in two households.

**Conclusions:**

The identification of identical *E*. *coli* strains containing the plasmid-mediated *mcr-1* gene in companion animals and humans in daily close contact is of concern. These results demonstrate the importance of the animal–human unit as possible disseminators of clinically important resistance genes in the community setting.

## Introduction

With the increasing trends of multidrug-resistant (MDR) pathogens worldwide, the use of colistin has emerged as one of the last-resort therapeutic options [1]. Until 2015, colistin resistance mechanisms were only due to chromosomal mutations. The emergence of the plasmid-mediated colistin resistance gene (*mcr-1*) changed this scenario [2]. Initially, the *mcr-1* gene was described in *Escherichia coli* strains from food-producing animals, retail meat and humans in China [2]. Then, it further spread globally among different Enterobacterales strains in sewage and river water, food (meat and vegetables), farm and wild animals, companion animals and humans (colonised and infected) [3-6]. This gene was confirmed to provide adequate phenotypical resistance against colistin treatment during its in vivo expression in a murine infection model. Furthermore, as the *mcr-1* gene can spread rapidly by horizontal transfer, this poses a notable public health concern [2].

Since the identification of *mcr-1*, nine additional *mcr* genes (*mcr-2* to *mcr-10*) have been described [7-9], with reports in the human, animal, and environmental settings worldwide [10]. The close contact between humans and companion animals increases the risk of resistant bacteria and/or gene transmission, raising issues for human health [11]. Possible dissemination of *mcr-1-*positive strains between companion animals and owners into households has been described in China and Ecuador [12,13].

Portugal has one of the highest consumptions of colistin in food-producing animals in Europe, as well as an intensive therapeutic colistin usage in humans [14]. These factors could explain the high prevalence of *mcr-1* gene that has been observed in food-producing animals and related products, as well as sporadic reports of detection in hospital inpatients [15,16]. However, in the community setting, no data are available on the role of companion animals and humans in the dynamic of transmission of colistin resistance.

In this longitudinal study, we aimed to identify the frequency and molecular characteristics of colistin-resistant *E*. *coli* from healthy companion animals and animals under antibiotic treatment for skin and urinary tract infections and their cohabiting humans in the community in Lisbon, Portugal by using whole genome sequencing (WGS). 

## Methods

### Study design, setting and participants

This prospective longitudinal study was conducted at the small animal veterinary teaching hospital of the Faculty of Veterinary Medicine, University of Lisbon, Portugal, between January 2018 and December 2020. This is a reference and first opinion hospital; referral consultations and second opinion consultations are held at an average of 100 attendances per day. 

Companion animals (dogs and cats) were enrolled in the study by convenience sampling; no active recruitment was performed. Animals from the Lisbon area that presented at the veterinary hospital for either well visits or care for infection were invited to participate in the study by the attending veterinarian. The companion animals and their cohabitating humans/owners from the same household were included in the study upon owners’ consent to participate. Other family members in the same household were also able to participate. Recruitment for the study was concluded when 40 households per group was achieved.

After examination, companion animals and humans were enrolled in the study. Questionnaires assessing demographic and general animal and human health data, previous medical treatment and exposure to hospital environment were performed. In addition, owners were asked about the animal's living environment and their contact with other animals. The owner questionnaire also inquired about their own travel history. For all variables on the questionnaire, the option ‘Prefer not to answer’ was available; the number of answers collected for any specific factor depended on whether the owner decided to disclose the information.

A total of 102 companion animals and 125 humans from 80 households were recruited. The household composition varied in the number of companion animals and humans (up to five humans and companion animals per household). To ensure that participation was anonymous, households, humans and animals were coded.

Two study groups were formed consisting of a control and an infection group, based on the health status of the animal (healthy vs under antibiotic use for mild infection). Groups were decided after the owner and animal questionnaires were completed (see Supplementary Figure S1 for the flow chart of households’ participants by study group). 

Inclusion criteria for enrolment of humans in this study were: (i) no systemic antimicrobial therapy in the last 3 months, (ii) no topical antimicrobial therapy in the 2 days before sampling (iii) living in the same household as included companion animals for at least 3 months, i.e. a cohabiting human. 

The control group was constituted by healthy dogs and cats and their cohabiting humans from 40 households. Companion animals were evaluated by their assistant veterinarians regarding their health status, and only healthy animals were enrolled in the control group. Other inclusion criteria for enrolment of animals in control group were all three (i─iii) mentioned above for the human participants. Cohabitating humans were also enrolled in the control group, including those who were healthy and those with chronic diseases, e.g. allergic, autoimmune and other conditions.

Animals were included in the infection group if they fulfilled the criteria for diagnosis of the following infections: urinary tract infection (UTI) according to the International Society of Companion Animal Infectious Diseases (ISCAID) guidelines [17], skin and soft tissue infection (SSTI) according to results of diagnostic tests (e.g. cytology and/or culture) and typical clinical signs of superficial pyoderma, deep pyoderma and wound infections. Other inclusion criteria for enrolment of companion animals with infection in this study group were only the absence of systemic antimicrobial therapy at the time of the veterinary appointment, i.e. criterion (i) above. Humans (healthy or with chronic diseases) cohabiting in the same household with animals were also enrolled in the infection group.

All enrolled dogs and cats from the infection group were prescribed first and/or second line antibiotics, according to the small animal veterinary teaching hospital antibiotic therapy internal operating procedures. These comply with the European Medicine Agency categorisation of antibiotics for prudent and responsible use in animals [18].

### Sample collection

At home, cohabiting humans collected partial faecal samples (that did not touch the ground) from their respective companion animals using sterile gloves and placed them into a sterile container. Humans collected their own faecal samples in sterile containers. Instructions for sample collection and storage at 4°C were given to the owners by a veterinary nurse. Samples were stored for a maximum of 48 h at 4°C until processing at the antibiotic resistance laboratory of the Faculty of Veterinary Medicine, University of Lisbon, Portugal.

In the control group, repeated sampling was performed monthly for 3 months (upon recruitment (T0), after 1 month (T2) and after 2 months (T3), (Table 1). Acquisition of follow-up samples depended on the owner’s willingness to continue to participate in the study with their respective companion animal. Antibiotic intake either by the human or the animal during the follow-up period resulted in exclusion from the study. For these reasons, at T2, sample collection was performed only for 19 households and, at T3, for 9 households. For graphical overview of sampling by timepoint, see Supplementary Figure S1.

**Table 1:** Data collection timepoints for the longitudinal study, Lisbon, Portugal, 2018–2020

Data collection timepoints	Control^a^ (n = 40 households)		Infection^b^ (n = 40 households)	
Timepoints	Animals (n = 82)	Humans (n = 56)	Animals (n = 40)	Humans (n = 69)
	n	n	n	n
T0: recruitment	82	56	34	59
T1: antibiotic treatment^c,d^	NA	NA	16	33
T2: 1 month after T0^e^	32	29	15	30
T3: 2 months after T0	13	13	11	21

NA: Not applicable; SSTI: skin and soft tissue infection; UTI: urinary tract infection.

^a^ In total, 40 control households (covering 42 dogs, 20 cats and 56 humans) were enrolled.

^b^ Forty households with SSTI or UTI animals (covering 35 dogs, 5 cats and 69 humans) were studied.

^c^ T1 was performed 1 week after antimicrobial treatment started.

^d^ For six households in the infection group (6 animals and 10 humans), recruitment started on this timepoint.

^e^ Two dogs from the infection group did not receive antimicrobial treatment (cases of superficial pyoderma secondary to atopy and an asymptomatic UTI, respectively) and sampling was not performed at T1.

Sample collection was scheduled at four timepoints for the infection group: before animal antimicrobial treatment (T0), 1 week after antimicrobial treatment started (T1), 1 month after antimicrobial treatment started (T2) and 2 months after antimicrobial treatment started (T3). Furthermore, as follow-up samples rested on owner’s/cohabiting human’s willingness to collaborate with the study, at T1 sample collection was performed only for 16 households, 15 for T2, and in 11 households for T3. Additionally, another reason for exclusion was the antibiotic intake by the person.

### Sample processing

One gram of homogenised faecal sample was added to 10 mL of sterile buffered peptone water (Biokar diagnostics) and plated onto SuperPolymyxin medium [19], an eosin methylene blue agar (Biokar diagnostics) supplemented with 3.5 μg/ml colistin (Sigma-Aldrich), 10 μg/ml daptomycin (Glentham Life Sciences), and 5 μg/ml amphotericin B (Glentham Life Sciences), and incubated for 24 h at 37°C. For each faecal sample, up to five colonies with a *E*. *coli* phenotype were isolated (i.e. metallic green sheen in SuperPolymyxin medium).

### Antimicrobial susceptibility testing

Minimum inhibitory concentrations (MICs) for colistin were determined for all *E*. *coli* isolates by broth microdilution (Sensititre FRCOL, Thermo Fisher Scientific), according to the manufacturer's instruction. Antimicrobial susceptibility testing for amikacin, amoxicillin/clavulanate, ampicillin, cefepime, cefotaxime, cefoxitin, ceftazidime, ciprofloxacin, chloramphenicol, gentamicin, meropenem and sulfamethoxazole/trimethoprim and were performed with MicroScan Neg MIC Panel Type 44 (Siemens) and interpreted according to the recommendations of the European Committee on Antimicrobial Susceptibility Testing (EUCAST) 2021 [20].

### Molecular characterisation of isolates

Bacterial DNA was extracted by heat lysis and centrifugation for all obtained isolates and *E*. *coli* strains were identified by PCR, as previously described [21,22].

All isolates were screened for nine mobile colistin resistance genes (*mcr-1* to *mcr-*9) by specific PCRs with subsequent sequencing of the amplified products by conventional sequencing technology [7,8]. Colistin-resistant isolates were also screened for the presence of extended-spectrum beta-lactamase (ESBL) genes (*bla*_CTX-M,_ *bla*_TEM_, *bla*_SHV_), plasmid-encoded AmpC (p*AmpC*) beta-lactamase genes (*bla*_DHA_, *bla*_CIT_, *bla*_LAT_, *bla*_ACT_, *bla*_MIR_, *bla*_FOX_, *bla*_MOX_), and carbapenemase beta-lactamase genes (*bla*_IMP_, *bla*_OXA_, *bla*_VIM_, *bla*_NDM_ *_,_ bla*_BIC,_ *bla*_SPM,_ *bla*_AIM,_ *bla*_GIM,_ *bla*_SIM,_ *bla*_DIM_ and *bla*_KPC_) [23,24]. Colistin-resistant isolates were further typed by multilocus sequence typing (MLST) according to the published consensus MLST scheme [25].

### Whole genome sequencing

Six *mcr-1*-positive isolates were subjected to WGS. This subset comprised two *E. coli* isolates arbitrarily selected (one dog and one human from different households) and four isolates from two co-colonised dog–human pairs from different households. DNA was extracted from RNase-treated lysates via NZY Tissue gDNA Isolation kit (NZYTech). Libraries for WGS were prepared using the TruSeq DNA PCR-Free preparation kit (Illumina). DNA sequencing was performed using Illumina NovaSeq platform with 2 x 150 bp paired-end reads at a private company (Macrogen, Seoul, Republic of Korea). An average 154x depth per strain was obtained.

Raw reads quality was analysed using FastQC v0.11.5 [26], adapters were trimmed using Fastx-Toolkit Clipper v0.0.13 [27] and the read quality filter was performed using PRINSEQ v0.20.4 [28] by selecting reads with a mean base quality score of ≥ 20 and minimum read length of 90 nucleotides. Draft de novo genome assemblies were generated using SPAdes v3.14.1 [29] followed by two runs of polishing with Pilon v1.24 [30]. An average N50 of 109 kb was obtained. Parsnp v1.2 [31] was used for multiple alignment of core genomes using *E*. *coli* K-12 MG1655 as a reference genome. Genealogies Unbiased By recomBinations In Nucleotide Sequences (Gubbins) was used to generate a phylogeny of core genomes corrected for recombination events with bootstrapping (100 replicates) [32]. The obtained tree and multiple alignment corrected for recombination were inputted into Raxml-NG in order to infer bootstrap support [33]. The Microreact platform [34] was used to visualise the phylogenetic tree linked to antimicrobial resistance data.

Putative antimicrobial resistance genes and mutations, as well as *E. coli* serotype virulence factors and plasmid replicon types were identified using ResFinder 4.0, SerotypeFinder 2.0, VirulenceFinder 2.0, PlasmidFinder 2.1 and pMLST 2.0 tools available at the Center for Genomic Epidemiology [35]. In silico detection of the insertion sequence IS*Apl1* (ISfinder) was also performed [36]. Alignment and visualisation of plasmids was performed with BRIG v0.95 [37]. Sequenced *E*. *coli* strains were deposited in the European Nucleotide Archive (ENA), short-read archive, project number PRJEB45751.

### Statistical analysis

The Fisher’s Exact test was used for comparisons between control and infection groups, with statistically significant difference at the p value < 0.05 level, using SAS statistical software package for Windows, version 9.3 (SAS Institute Inc).

## Results

### Epidemiological survey and study enrolment

A total of 125 humans and 102 cohabitating animals from 80 households were enrolled in a prospective longitudinal study between January 2018 and December 2020 (see Supplementary Figure S1 for a flow chart of households’ participants by study group). The control group was constituted by healthy dogs (42/62) and cats (20/62) from 40 households. Cohabitating humans (n = 56) were also enrolled in the control group, including those who were healthy (35/56) and those with chronic diseases (21/56). The infection group included dogs (35/40) and 5 cats (5/40) with UTI (n = 18), SSTI (n = 22) and cohabitating humans (n = 69), either healthy (42/69) or with chronic diseases (27/69). Participants demographic, clinical and social data retrieved at collection point T0 are summarised in Table 2.

**Table 2:** Questionnaire responses on demographic, social and clinical characteristics of humans (n = 125) and companion animals (n = 102) by study group, Lisbon, Portugal, 2018–2020

Characteristics	Colonised participants	Control group (n/N)	Infection group (n/N)
Demographic^a^			
Female	Humans	42/56	42/69
	Dogs	20/42	17/35
	Cats	9/20	3/5
Male	Humans	14/56	27/69
	Dogs	22/42	18/35
	Cats	11/20	2/5
Mean age (range) in years, (n)	Humans	35.5 (6–67) (n = 56)	43.1 (3–77) (n = 67)
	Dogs	6.7 (0.25–17) (n = 35)	8.1 (1.8–15) (n = 34)
	Cats	9.1 (1–15) (n = 16)	11 (5-15) (n = 5)
Clinical^b^			
Hospitalisation within 12 months of sampling	Humans	5/54	7/67
	Dogs	6/42	11/34
	Cats	2/20	3/5
Systemic antimicrobial treatment within 12 months of sampling	Humans	16/55	28/58
	Dogs	9/42	28/34
	Cats	5/20	4/5
Systemic antimicrobial treatment within 3–6 months of sampling	Humans	3/55	16/58
	Dogs	3/42	10/34
	Cats	1/20	3/5
Social			
Indoor lifestyle	Dogs	30/42	31/35
	Cats	20/20	5/5
Sleeps in human bed^c^	Dogs	19/42	14/34
	Cats	16/20	3/5
Socialised with other animals outside the household^c^	Dogs	23/42	16/34
	Cats	0/20	0/5
Boarding pet hotel within 12 months of sampling^c^	Dogs	7/42	6/34
	Cats	0/20	0/5
Other			
Healthcare professional^d^	Humans	20/56	8/67
Travel outside Europe within the past 12 months	Humans	9/56	11/67

NA: not applicable.

^a^ Missing data regarding age for seven dogs and four cats from control group, one dog and two humans of infection group.

^b^ Antimicrobial treatment information was retrieved at the first collection point T0 (before animal antimicrobial treatment for infections group). Hospitalisation data was missing for two humans from control group and two humans and one dog from infection group. Systemic antimicrobial treatment data missing for one human from control group, 11 humans and one dog from infection group.

^c^ Social data information missing for one dog from the infection group.

^d^ Including human health care and animal health care.

Two dogs from the infection group (n = 40) did not receive antimicrobial treatment (cases of superficial pyoderma secondary to atopy and an asymptomatic UTI, respectively) and sampling was not performed at T1 (see Supplementary Figure S1). For six animals, it was not possible to perform the first collection point (T0, before the antimicrobial treatment) in due time and sample collection started at T1.

Companion animals’ ages ranged from 3 months to 17 years (median: 7 years), and humans were aged 3 to 77 years (median: 39). Eight companion animals and five humans from the control group had been hospitalised within the 12 months prior to the first sample. Fourteen companion animals and seven humans from the infection group were hospitalised 12 months prior to sampling (Table 2).

### Frequency of colistin-resistant *Escherichia coli* strains 

**Figure 1:**
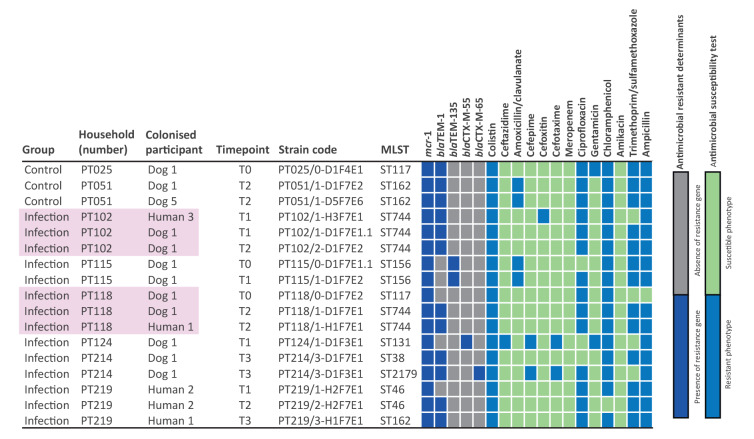
Genotypic and phenotypic characteristics of colistin-resistant *Escherichia coli* isolates obtained from faeces of eight dogs and four humans, Lisbon, Portugal, 2018–2020 (n = 17 isolates)

Seventeen *E. coli* strains collected at different timepoints were obtained from eight dogs (n = 3 healthy, n = 4 SSTI, n = 1 UTI) of 102 companion animals (7.8%, 95% CI: 2.5–13.1) and four of 125 humans (3.2%, 95% CI: 0.07–6.3). All the colistin-resistant *E. coli* strains isolated from humans belonged to the infection group households. MICs confirmed reduced susceptibility to colistin for these 17 *E. coli* isolates (range: 2–8 mg/L), all of them presenting a multidrug-resistant profile (Figure 1, see Supplementary Table S1 for the minimum inhibitory concentrations for colistin-resistant *E. coli* strains). There was no significant difference between the frequency of colonisation by colistin-resistant *E. coli* in animals from the control and infection groups (p = 0.257). 

MLST: Multilocus sequence typing.

Shaded boxes highlight the households with common strains. Colonised participant number refers to participant codification within the household. The heatmap shows the presence or absence of antimicrobial resistance genes and minimum inhibitory concentration interpretation results (see colour key on the right side of the figure). See Table 1 for further explanations regarding collection timepoints.

Of the individuals colonised by colistin-resistant *E*. *coli*, co-carriage by dog and owner was observed in two households from the infection group (PT102 and PT118).

MLST typing identified eight sequence types (ST117, ST162, ST744, ST156, ST131, ST2179, ST38 and ST46) among the 17 colistin-resistant isolates (Figure 1). ESBL *bla*_CTX-M-55_ and *bla*_CTX-M-65_ genes were found in one *E*. *coli* ST131 strain and in one *E*. *coli* ST2179 strain, respectively, from different animals. The *bla*_TEM-1_ gene was the most frequently found resistance gene after *mcr-1*, being present in 12 isolates from 10 different individuals. No p*AmpC* or carbapenemase beta-lactamase-encoding genes were found (Figure 1).

### Longitudinal *mcr-1* gene carriage among dogs and cohabiting humans

**Figure 2:**
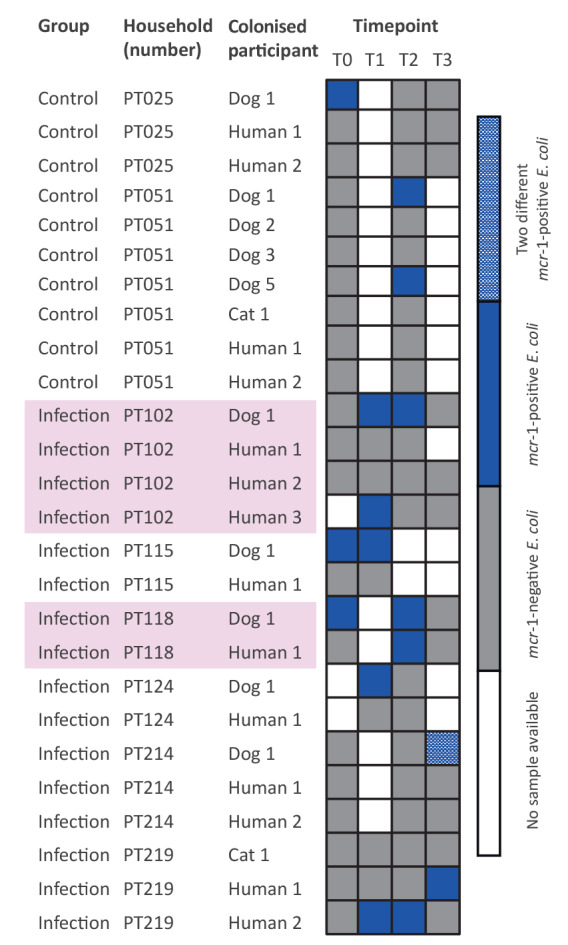
Distribution of *Escherichia coli* harbouring the *mcr*-1 gene across faecal sampling timepoints in 12 animals and 14 cohabitating humans, Lisbon, Portugal, 2018–2020 (n = 8 households)

Of the 40 households enrolled in the control group, samples from 19 households after 1 month (T2) and from nine households after 2 months (T3) were received (see Supplementary Figure S1), as the collection of follow-up samples was dependent on owners who were willing to continue to participate in the study. Eight dogs, five cats and 13 humans had samples at all three timepoints (T0, T2 and T3). Of the two households in the control group, only one household (PT025) with a dog carrying *mcr-1*-positive *E*. *coli* at T0 had samples at all three timepoints (Figure 2). Another household (PT051) of the control group had two dogs carrying *mcr-1*-positive *E*. *coli*, both of which had samples at T0 and T2, but positive isolates were only recovered at the second timepoint (Figure 2). 

Shaded boxes highlight the households with common strains. Colonised participant number refers to participant codification within the household. Control group: healthy companion animals and their cohabiting humans. Infection group: companion animals with urinary tract or skin and soft tissue infections and their cohabiting humans. See Table 1 for further explanations regarding collection timepoints.

From 40 households of the infection group, only six presented individuals carrying *mcr-1*-positive *E*. *coli* strains. Two households (PT102 and PT219) had samples at all timepoints, and the other five provided samples for at least two consecutive timepoints (Figure 2). For two dogs and one human from different households (PT102, PT115 and PT219, respectively), *mcr-1*-positive *E*. *coli* were recovered in two consecutive timepoints (Figure 2). These repetitive strains were typed to the same MLST and presented the same genetic environment over time (Figure 1).

### Genetic relatedness of *mcr-1* strains

**Figure 3:**
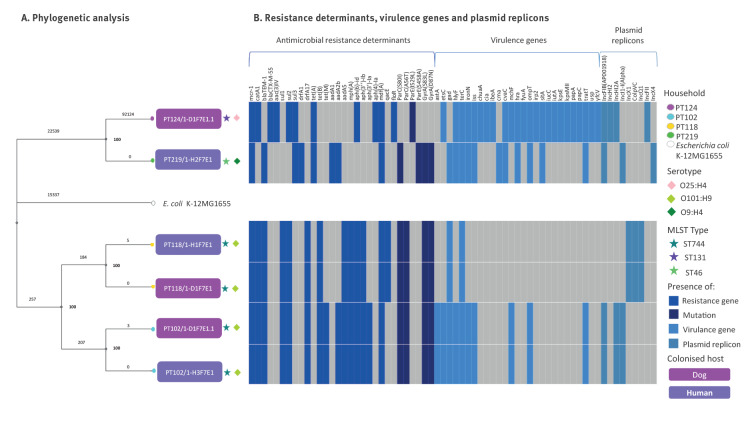
Relatedness of *Escherichia coli* strains from animals and cohabitating humans and corresponding genetic features, Lisbon, Portugal, 2018–2020 (n = 6)

Analysis of the genetic relatedness of the core genomes of obtained assemblies from *E. coli* strains, also aligned with *E. coli* K12 MG1655, showed that genomes from four strains of cohabiting dogs and humans displayed a reduced number of single nucleotide polymorphisms (SNPs) and were allocated inside the same cluster, suggesting transmission within household or a common source of infection (Figure 3). These paired strains presented the same virulence and resistance genes and belonged to ST744, serotype O101:H9 (Figure 3).

MLST: Multilocus sequence typing.

A. Maximum likelihood phylogeny of the core genome of six *E. coli* isolates and *E. coli* K-12 MG1655. Bootstrap support values are shown in bold. Total number of SNPs are indicated on each arm.

B. Heatmap showing the antimicrobial resistance determinants, virulence genes and plasmid replicons for each isolate (see colour key on the right side of the figure).

One strain from a dog (PT124/1-D1F3E1) belonged to the pandemic human *E*. *coli* ST131 lineage, carrying the *mcr-1* and *bla*_CTX-M-55_ genes. This strain exhibited a combination of virulence factors that classified it as an extraintestinal pathogenic *E*. *coli* (ExPEC), namely, the presence of P fimbriae (*papC*) and aerobactin siderophore (*iutA*) (see Supplementary Table S2 for the genomic features display on whole genome sequencing of colistin‑resistant *E*. *coli* strains, Figure 3) [38]. Although the cohabiting human carried a colistin-susceptible *E*. *coli* ST131 isolate, it did not harbour the *mcr-1* gene (data not shown).

**Figure 4:**
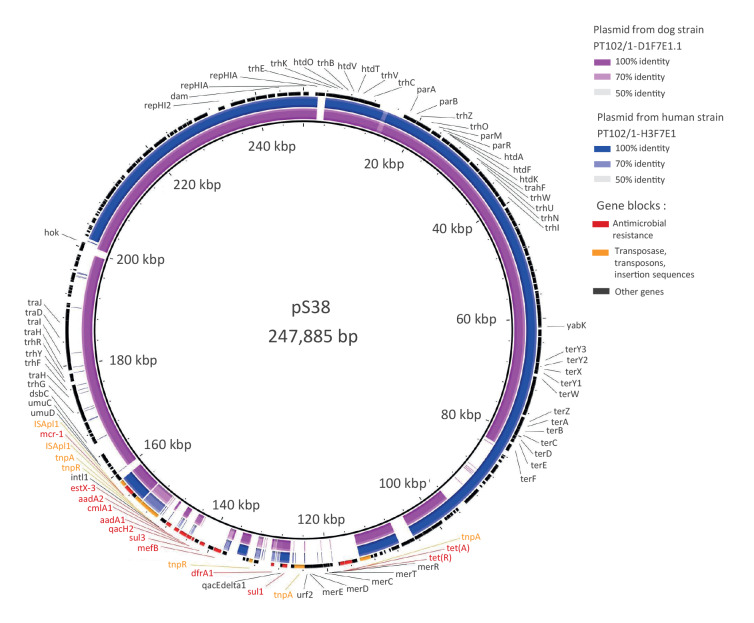
Alignment of *mcr-1-*carrying plasmids from shared *Escherichia coli* strains from a dog and its cohabiting human, Lisbon, Portugal, 2018–2020

In addition to the colistin-resistant *mcr-1* gene, all the sequenced strains exhibited a wide variety of genes encoding resistance against phenicols (*catA1*, *cmlA1, floR*), beta-lactams (*bla*_TEM–1_, *bla*_CTX-M-55_), sulphonamides (*sul1*, *sul2*, *sul3*), trimethoprim (*dfrA1*, *drfA17*), tetracyclines (*tet*(A), *tet*(B)), macrolides (*mph*(A), *mdf*(A)), aminoglycosides (*aadA5*, *aph(6)-id, aph(3’)-Ia, aph(3”)-Ib, aac(3)-IV aph(4)-Ia*, *aadA1*, *aadA2b*), as well as point mutations in *gyrA*, *parC*, and/or *parE* genes of the II and IV topoisomerases, respectively, which could confer resistance to nalidixic acid and ciprofloxacin (Figure 3, Supplementary Table S2). Sequenced isolates harboured multiple plasmid replicons from different plasmid incompatibility groups (Figure 3, Supplementary Table S2). The shared human-dog *E*. *coli* ST744 strains in households PT102 and PT118 presented the same plasmid replicons (IncFIB (AP001918), IncHI2A and IncI1-I(Alpha) or ColpVC, IncQ1 and IncX1, respectively). For strain PT219/1-H2F7E1, the *mcr-*1 gene was observed in the same contig as plasmid replicon IncX4. Regarding PT102/1-D1F7E1.1 and PT102/1-H3F7E1 *E*. *coli* ST744 strains, the plasmid replicon IncHI2A, the insertion sequence element IS*Apl1* and the colistin resistance *mcr-1* gene were found in the same contig. Comparison with plasmid pS38 harbouring the *mcr*-1 gene (GenBank accession number KX129782.1) made possible the partial reconstruction of a putative plasmid pPT102D1H3 (Figure 4). These IncHI2-type plasmids from the PT102 household strains were assigned to pMLST ST4 type. Plasmid reconstructions were based on short-read sequence data (Illumina). This technology does not allow for a high-quality assembly of the plasmids, as these mobile elements present a high number of repeated sequences, and so it was not possible to establish the circular plasmid nucleotide sequence for the strains where *mcr-1* gene and the plasmid replicons were not found in the same contig.

Alignment of PT102/1-D1F7E1.1 (dog) and PT102/1-H3F7E1 (human) assembled sequences against plasmid pS38 (GenBank accession number KX129782.1). Black inner ring: plasmid used as reference for the alignment; name and size of the reference indicated in the middle of the panel. Genes are represented by coloured blocks in the outer circle (colour key on the right side of the figure).

## Discussion

In the present longitudinal study, we assessed the frequency of colonisation by colistin-resistant *E*. *coli* harbouring the *mcr*-*1*-plasmid-mediated gene in companion animals and humans. A control group (healthy companion animals) and an infection group (animals with UTI and SSTI under antibiotic therapy), and their cohabiting humans were studied to evaluate the effect of antibiotic usage in companion animals on the frequency of colonisation by colistin-resistant *E*. *coli*. Yet, our results did not show a significant difference between the frequency of colonisation/carriage by colistin-resistant *E. coli* in animals from the control and infection groups.

Our study revealed a frequency of colonisation/carriage by *mcr*-*1*-positive *E. coli* strains of 7.8% in dogs and 3.2% in humans in the community from the Lisbon region. The unexpectedly high frequency of *mcr-1* in *E*. *coli* strains from dogs highlights the potential that dogs have as a reservoir and consequently the importance of the human–companion animal relationship in the dissemination of this resistance determinant. Faecal colonisation by *mcr-1-*positive strains has been detected among companion animals in Asia and South America [12,13,39]. To the best of our knowledge, there is only one report in Europe, on a barn dog of a pig farm [40]. Our results agree with the findings of a Chinese study where 8.7% of companion animals harboured *mcr-1* in Enterobacterales [39]. 

The proportion of *mcr-1* carriers among human participants (3.2%) was relevant as findings on faecal carriage of the *mcr-1* gene in Europe have been associated to travellers returning from countries outside Europe [3]. Yet, in the present study, none of the individuals carrying the resistance determinant travelled outside of Europe in the 12 months prior to sample collection. The hospital setting has also been strongly associated with the epidemiology of the *mcr-1* gene in humans, as was reported in Portuguese inpatients [15,16]. Here, only one of the four human *mcr-1-*positive participants was hospitalised in the 12 months before sampling, and none were health professionals.

The colonisation of *mcr-1-*positive *E*. *coli* strains over time was only observed in three of the 12 colonised hosts (one dog with a 1-week interval, another dog and one human in 2 consecutive months), indicating transient colonisation in most of the cases. However, as we did not sequence the recurrent strains by WGS to compare them, we cannot say that they are similar.

All the *mcr-1*-positive isolates detected in the study presented an MDR profile, 4/17 of the isolates co-produced ESBL enzymes, and 12/17 co-produced narrow-spectrum beta-lactamase (*bla*_TEM–1_). This is worrisome, particularly in light of the potential of *mcr-1* to coexist with other resistance genes on the same plasmid, as co-selection may occur regardless of colistin usage. 

The virulent high risk clonal lineage ST131 was isolated from companion animal faeces; this successful, highly disseminated clone has already been reported to carry the *mcr-1* gene worldwide [38,41]. Of the eight colistin-resistant *E*. *coli* lineages detected in this study, only ST744 and ST162 were common to both animals and humans. The *E*. *coli* ST162 lineage has already been identified at the human–environment–animal interface worldwide, indicating that the *mcr-1* gene could potentially be disseminated through this *E*. *coli* lineage across these One Health settings [42]. The *E*. *coli* ST744 lineage has demonstrated high potential for *mcr* gene dissemination by its association with the transmission of *mcr-1-*positive strains across abattoirs in Romania [43]. In Portugal, this lineage co-harbouring *mcr-1* and *bla*_KPC-3_ genes was previously detected in a urine culture from an inpatient [16]. In the present study, we detected four *E*. *coli* ST744 strains harbouring the *mcr-1* gene that were shared between dogs and humans in two of 80 households from the Lisbon region. The two paired core genomes sequences differed by less than six SNPs, proving the sharing of these *mcr-1-*positive *E*. *coli* ST744 strains between animals and humans living together.

The *mcr-1* gene mobilisation was found to be associated to IncHI2-type subtype ST4 plasmids in the two shared *E*. *coli* ST744 strains. This particular plasmid harbouring the *mcr-1* gene, is found to be widespread though European farm animals, highlighting its potential on the successful dissemination of this clinical important gene into the community [41]. A mobile transposon element, IS*Apl1,* was also detected in these two shared *E*. *coli* ST744 strains. This element has been shown to play a strong role in the mobility of the *mcr-1* gene [10,44]. According to a recent study, an initial mobilisation of this resistance determinant by the IS*Apl1* transposon element occurred in the mid-2000s, followed by the loss of the flanking Insertion Sequence (IS) on several plasmid backgrounds because of high instability, which contributed to the retention of the *mcr-1* gene in the plasmids and to its spread [44]. This phenomenon could explain the two different groups of *E*. *coli* ST744 observed.

Several studies have reported the colonisation and sharing of Enterobacterales strains and/or antimicrobial resistance determinants between companion animals and humans. Here, we report two paired similar core genomes sequences of commensal *mcr-1*-positive *E. coli* strains in dogs and humans from the Lisbon region. Additionally, the *E*. *coli* ST744 strains from one household presented the *mcr-1* gene and an IS*Apl1* in a similar IncHI2-type ST4 subtype plasmid. As such, households might constitute an epidemiological unit to be considered in the efforts to combat the spread of this important resistance determinant. 

A primary limitation of this study is the small number of subjects per study group, which was not powered to detect changes. In particular, the longitudinal study relied on the owners/cohabiting human willingness to take part in the study with their respective companion animal. Additionally, another reason for exclusion was the antibiotic intake either by the person or the animal. Due to the small number of participants, we were not able to identify specific risk factors, i.e., recent hospitalization or cohabiting with a colonised subject, for *mcr-1* carriage in the present study. Another limitation was the challenge to fully characterise all the plasmids harbouring the *mcr-1* gene. 

## Conclusions

This study has shown the importance of the animal–human epidemiological unit in the community, as similar *E*. *coli* strains containing the plasmid-mediated *mcr-1* gene were described in dogs and humans in daily close contact. An interdisciplinary collaboration in a One Health perspective is critical to create strategies to mitigate the transmission of plasmid-mediated colistin-resistant strains among humans and companion animals. Considering that the use of polymyxins in veterinary medicine, livestock and human medicine exerts a selective pressure for the emergence of plasmid-mediated colistin-resistant strains, an active control of this antimicrobial usage is urgently needed to mitigate the spread of resistance to other bacterial species in the community, the environment and hospital facilities.

## Supplementary Data

226000 Supplement
