# Supplementary material for: mcr-1 colistin resistance gene sharing between Escherichia coli from cohabiting dogs and humans, Lisbon, Portugal, 2018 to 2020
Source: Euro Surveill. 2022 Nov 3;27(44):2101144. doi: 10.2807/1560-7917.ES.2022.27.44.2101144 (PMC9635019; doi:10.2807/1560-7917.ES.2022.27.44.2101144)
Supplement: Supplement [file 21-01144_POMBA_SUPPLEMENT.pdf]

mcr-1 colistin resistance gene sharing between *Escherichia coli* from cohabiting dogs and humans, Lisbon, Portugal, 2018 to 2020

Supplementary material

This supplementary material is hosted by *Eurosurveillance* as supporting information alongside the article "*mcr-1* colistin resistance gene sharing between *Escherichia coli* from cohabiting dogs and humans, Lisbon, Portugal, 2018 to 2020" on behalf of the authors, who remain responsible for the accuracy and appropriateness of the content. The same standards for ethics, copyright, attributions and permissions as for the article apply. Supplements are not edited by *Eurosurveillance* and the journal is not responsible for the maintenance of any links or email addresses provided therein.

Table S1. Minimum inhibitory concentrations for colistin-resistant *Escherichia coli* strains isolated from faeces samples of dogs and co-habiting humans

Group	Strain code	Household (number)	Colonised participant[a]	Minimum inhibitory concentration (mg/L)												
				AK	AMC	AMP	C	CAZ	CIP	CPM	CS	CTX	FOX	GEN	MEM	SXT
Control	PT025/0-D1F4E1	PT025	Dog 1	≤8	≤8/4	>**16**	>**16**	≤1	>**2**	≤1	**4**	≤1	≤8	>**8**	≤1	>**4/76**
Control	PT051/1-D1F7E2	PT051	Dog 1	≤8	**16/8**	>**16**	>**16**	≤1	>**2**	≤1	**4**	≤1	≤8	≤2	≤1	>**4/76**
Control	PT051/1-D5F7E6	PT051	Dog 5	≤8	**16/8**	>**16**	>**16**	≤1	>**2**	≤1	**4**	≤1	≤8	≤2	≤1	>**4/76**
Infection	PT102/1-H3F7E1	PT102	Human 3	≤8	≤8/4	>**16**	>**16**	≤1	>**2**	≤1	**2**	≤1	**16**	≤2	≤1	≤2/38
Infection	PT102/1-D1F7E1.1	PT102	Dog 1	≤8	≤8/4	>**16**	>**16**	2	>**2**	2	**2**	≤1	≤8	≤2	≤1	>**4/76**
Infection	PT102/2-D1F7E2	PT102	Dog 1	≤8	≤8/4	>**16**	>**16**	≤1	>**2**	≤1	**4**	≤1	≤8	≤2	≤1	>**4/76**
Infection	PT115/0-D1F7E1.1	PT115	Dog 1	≤8	>**16/8**	>16	>**16**	≤1	≤0.5	4	**2**	≤1	≤8	≤2	≤1	>**4/76**
Infection	PT115/1-D1F7E2	PT115	Dog 1	≤8	**16/8**	>**16**	>**16**	≤1	>**2**	≤1	**2**	≤1	≤8	≤2	≤1	>**4/76**
Infection	PT118/0-D1F7E2	PT118	Dog 1	≤8	≤8/4	≤8	>**16**	≤1	**1**	≤1	**8**	≤1	≤8	≤2	≤1	≤2/38
Infection	PT118/1-D1F7E1	PT118	Dog 1	≤8	≤8/4	>**16**	>**16**	≤1	>**2**	≤1	**4**	≤1	≤8	≤2	≤1	>**4/76**
Infection	PT118/1-H1F7E1	PT118	Human 1	≤8	≤8/4	>**16**	>**16**	≤1	>**2**	≤1	**2**	≤1	≤8	≤2	≤1	>**4/76**
Infection	PT124/1-D1F3E1	PT124	Dog 1	≤8	≤8/4	>**16**	>**16**	>**16**	≤0.5	>**16**	**8**	>**32**	≤8	>**8**	≤1	≤2/38
Infection	PT214/3-D1F7E1	PT214	Dog 1	≤8	≤8/4	>**16**	>**16**	≤1	≤0.5	≤1	**4**	≤1	≤8	≤2	≤1	>**4/76**
Infection	PT214/3-D1F3E1	PT214	Dog 1	≤8	≤8/4	>**16**	>**16**	≤1	>**2**	>**16**	**4**	>**32**	≤8	≤2	≤1	≤2/38
Infection	PT219/1-H2F7E1	PT219	Human 2	≤8	≤8/4	>**16**	>**16**	≤1	>**2**	≤1	**4**	≤1	≤8	≤2	≤1	>**4/76**
Infection	PT219/2-H2F7E1	PT219	Human 2	≤8	≤8/4	>**16**	≤8	≤1	>**2**	≤1	**4**	≤1	≤8	≤2	≤1	>**4/76**
Infection	PT219/3-H1F7E1	PT219	Human 1	≤8	≤8/4	>**16**	>**16**	≤1	>**2**	≤1	**4**	≤1	≤8	≤2	≤1	>**4/76**

Bold text indicates *E. coli* strain classification as resistant according to clinical breakpoints set by EUCAST 2021 guidelines (https://www.eucast.org/fileadmin/src/media/PDFs/EUCAST_files/Breakpoint_tables/v_11.0_Breakpoint_Tables.pdf), except for amoxicillin/clavulanate and sulfamethoxazole/trimethoprim, for which criteria from the Clinical and Laboratory Standards Institute (CLSI) (Performance Standards for Antimicrobial Susceptibility Testing. 30th ed. CLSI supplement M100. Wayne, PA: Clinical and Laboratory Standards Institute; 2020) were used.
AK: amikacin; AMC: amoxicillin/clavulanate; AMP: ampicillin; C: chloramphenicol; CAZ: ceftazidime; CIP: ciprofloxacin; CPM: cefepime; CS: colistin; CTX: cefotaxime; FOX: cefoxitin; GEN: gentamicin; MEM: meropenem; MLST: multilocus sequence typing; SXT: sulfamethoxazole/trimethoprim.

[a]Colonised participant number refers to participant codification within the household

Table S2. Genomic features display on whole genome sequencing of colistin-resistant *Escherichia coli* strains

Strain code	Household (number)	Colonised participant	Virulence genes	Serotype	Plasmid's replicons	Antimicrobial resistant determinants		No. IS*Apl1*
						Acquired resistance genes	Chromosomal point mutations	
PT102/1-D1F7E1.1	PT102	Dog	*astA, etsC, gad, hlyF, ironN, iss, mchF, ompT, terC, traT*	O101:H9	IncFIB (AP001918), IncHI2A, IncI1-I(Alpha)	*mcr-1, catA1, bla*$_{TEM-1}$, *sul1, sul2, drfA12, drfA17, tet*(M), *tet*(B), *mph*(A), *aadA5, aadA2, aph(6)-id, aph(3')-Ia, aph(3")-Ib, mdf(A)*	ParC (S80I and A56T), GyrA (S83L and D87N)	1
PT102/1-H3F7E1	PT102	Human	*astA, etsC, gad, hlyF, ironN, iss, mchF, ompT, terC, traT*	O101:H9	IncFIB (AP001918), IncHI2A, IncI1-I(Alpha)	*mcr-1, catA1, bla*$_{TEM-1}$, *sul1, sul2, drfA12, drfA17, tet(M), tet(B), mph(A), aadA5, aadA2, aph(6)-id, aph(3')-Ia, aph(3")-Ib, mdf(A)*	ParC (S80I and A56T), GyrA (S83L and D87N)	1
PT118/1-D1F7E1	PT118	Dog	*gad, terC*	O101:H9	IncX1, ColpVC, IncQ1	*mcr-1, catA1, blaTEM*$_{-1}$, *sul1, sul2, drfA17, tet(B), mph(A), aadA5, aph(6)-id, aph(3")-Ib, mdf(A), qacE*	ParC (S80I and A56T), GyrA (S83L and D87N)	0
PT118/1-H1F7E1	PT118	Human	*gad, terC*	O101:H9	IncX1, ColpVC, IncQ1	*mcr-1, catA1, blaTEM*-1, *sul1, sul2, drfA17, tet(B), mph(A), aadA5, aph(6)-id, aph(3")-Ib, mdf(A), qacE*	ParC (S80I and A56T), GyrA (S83L and D87N)	0
PT124/1-D1F3E1	PT124	Dog	*chuA, cia, cvaC, etsC, fyuA, hlyF, hra, ibeA, iroN, irp2, iss, iucC, iutA, kpsE, kpsM* II, *mchF, ompT, papA, papC, sitA, terC, traT, usp, yfcV*	O25:H4	IncFIB (AP001918), IncFII, IncHI2, IncHI2A	*mcr-1, catA1, bla*$_{CTX-M-55}$, *aac(3)-IV, sul3, sul2, tet*(A), *aadA1, aph(6)-Ib, aph(4)-Ia, mdf(A), floR*	ParE (I529L)	0

Strain code	Household (number)	Colonised participant	Virulence genes	Serotype	Plasmid's replicons	Antimicrobial resistant determinants		No. IS*Apl1*
						Acquired resistance genes	Chromosomal point mutations	
PT219/1-H2F7E1	PT219	Human	*cma, cvaC, gad, hlyF, hra, iroN, iss, ompT, sitA, terC, traT*	O9:H4	IncFIB (AP001918), IncX4, IncI1-I (Alpha)	*mcr-1, cmlA1, bla*$_{TEM-1}$, *sul3, drfA1, tet*(A), *aadA1*, *aadA2b*, *mpf*(A)	ParC (S80I), ParE (S458A), GyrA (S83L and D87N)	0

astA: EAST-1 heat-stable toxin; *chuA: E.coli* hemeutilization protein A gene; *cia:* Invasion antigens; *cma:* Colicin M; *cvaC*, colicins; *etsC:* encode for Putative type I secretion outer membrane protein; *fyuA:* Ferrin yersiniabactin uptake; *gad:* Glutamate decarboxylase; *hlyF:* Hemolysin F; *hra:* heat-resistant agglutinin; *ibeA:* invasion brain endothelium; *ireA:* Siderophore receptor; *iroN:* Enterobactin siderophore receptor; *irp2:* Iron-repressible protein; *iss:* Increased serum survival; *iucC:* Aerobactin; *kpsE:* Transfer protein; *kpsM* II: Group 2 capsule synthesis; *mchF:* ABC transporter protein MchF; *iutA:* Aerobactin siderophore; *ompT: Outer membrane protein; papA: Structural subunit of P-fimbriae; papC: P fimbriae; sitA:* transporter protein; *terC:* Tellurite resistance; *traT:* Serum resistance associated gene; *usp:* gene encoding for uropathogenic specific protein; *yfcV:* Major subunit of a putative chaperone-usher fimbria.

Figure S1. Flow chart of households' participants by study group.

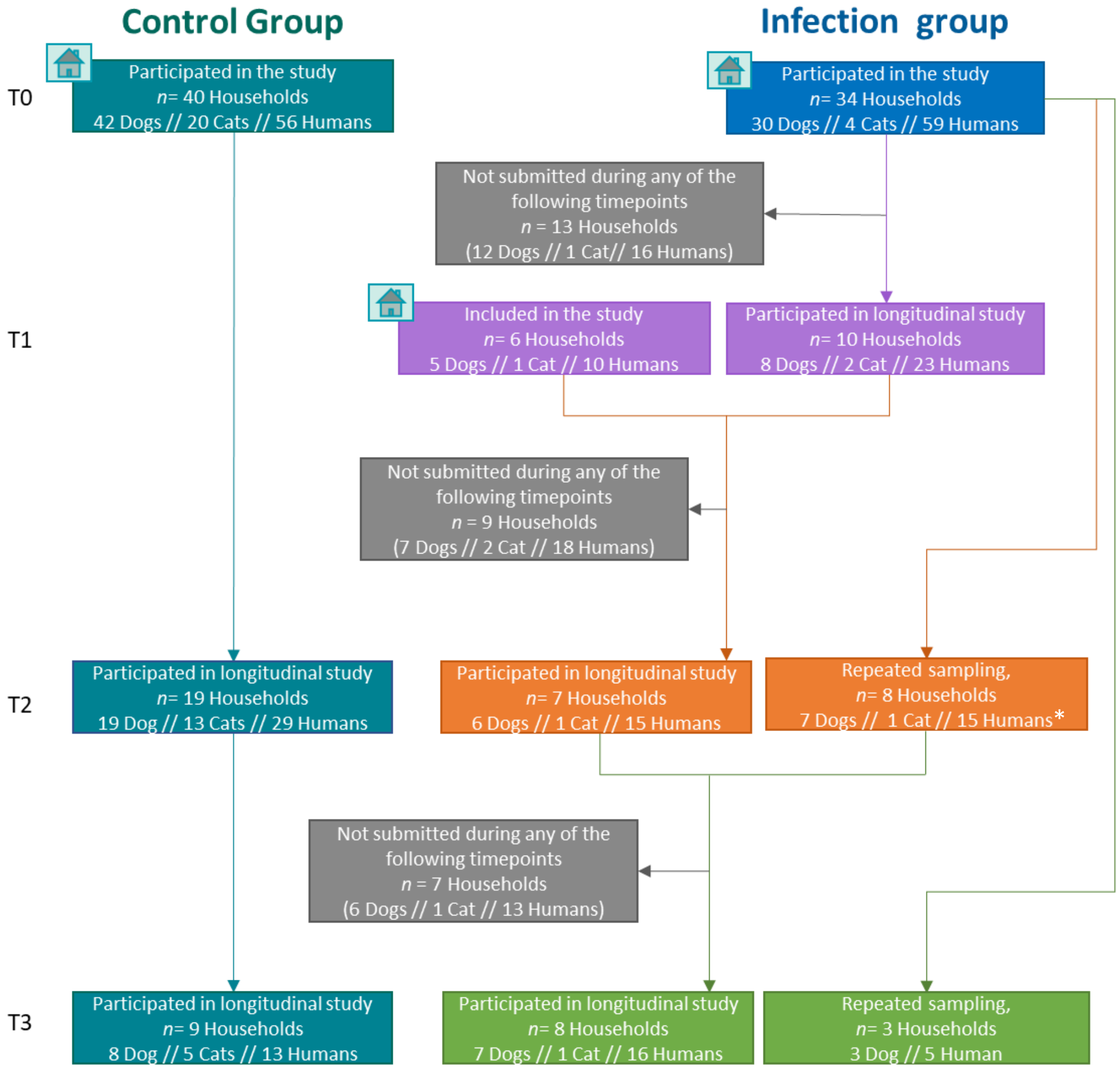

House symbol represents insertions of new households to the study. For the control group: T2 was performed one month after T0 sampling; T3 was done two months after T0. For infection group cases: T0 concerns the sampling before antimicrobial intake; T1 was done one week after antimicrobial treatment started; T2 one month after antimicrobial treatment started; T3 was achieved 2 months after antimicrobial treatment started; In total, 40 control households (covering 42 dogs, 20 cats and 56 Humans) and 40 households with SSTI or UTI animals (covering 35 dogs, 5 cats and 69 humans) were studied; *Two dogs from the infection group (n = 40) did not receive antimicrobial treatment (cases of superficial pyoderma secondary to atopy and an asymptomatic UTI, respectively) and sampling was not performed at T1. These dogs are included in this Repeated sampling Households' box.
